# Effects of periostin deficiency on kidney aging and lipid metabolism

**DOI:** 10.18632/aging.203580

**Published:** 2021-10-03

**Authors:** Jung Nam An, Hyoseon Kim, Eun Nim Kim, Ara Cho, Yeongeun Cho, Young Wook Choi, Jin Hyuk Kim, Seung Hee Yang, Bum Soon Choi, Chun Soo Lim, Yon Su Kim, Kwang Pyo Kim, Jung Pyo Lee

**Affiliations:** 1 Department of Internal Medicine, Hallym University Sacred Heart Hospital, Anyang, Gyeonggi-do, Korea; 2 Department of Applied Chemistry, Institute of Natural Science, Global Center for Pharmaceutical Ingredient Materials, Kyung Hee University, Yongin, Korea; 3 Department of Biomedical Science and Technology, Kyung Hee Medical Science Research Institute, Kyung Hee University, Seoul, Korea; 4 Department of Internal Medicine, College of Medicine, The Catholic University of Korea, Seoul, Korea; 5 Department of Internal Medicine, Seoul National University Boramae Medical Center, Seoul, Korea; 6 Department of Urology, College of Medicine, Chung-Ang University, Seoul, Korea; 7 Seoul National University Kidney Research Institute, Seoul, Korea; 8 Biomedical Research Institute, Seoul National University Hospital, Seoul, Korea; 9 Department of Internal Medicine, Seoul National University College of Medicine, Seoul, Korea; 10 Department of Internal Medicine, Seoul National University Hospital, Seoul, Korea

**Keywords:** periostin, kidney, aging, lipid, lipidomics

## Abstract

Periostin plays a crucial role in fibrosis, which is involved in kidney aging. A few studies have shown that lipid metabolism is involved in kidney aging. We investigated the role of periostin in lipid metabolism during kidney aging. Renal function, fibrosis, and inflammatory markers were studied using urine, blood, and tissue samples from wild-type (WT) C57BL/6 mice and *Postn-null* mice of 2 and 24 months of age. Lipids were quantitatively profiled using liquid chromatography-tandem mass spectrometry in the multiple reaction monitoring mode. Renal function was worse and tubular atrophy/interstitial fibrosis, periostin expression, and inflammatory and fibrotic markers were more severe in aged WT mice than in young WT mice. In aged *Postn-null* mice, these changes were mitigated. Thirty-five differentially regulated lipids were identified. Phosphatidylcholines, cholesteryl ester, cholesterol, ceramide-1-phosphate, and CCL5 expression were significantly higher in aged WT mice than in aged *Postn-null* mice. Particularly, linoleic acid, linolenic acid, arachidonic acid, and docosahexaenoic acid differed strongly between the two groups. Lysophosphatidylcholine acyltransferase 2, which converts lysophosphatidylcholine to phosphatidylcholine, was significantly higher in aged WT mice than in aged *Postn-null* mice. Periostin expression in the kidneys increased with age, and periostin ablation delayed aging. Changes in lipids and their metabolism were found in *Postn-null* mice. Further research on the precise mechanisms of and relationships between lipid expression and metabolism, kidney aging, and periostin expression is warranted.

## INTRODUCTION

The global population continues to grow rapidly, and life expectancy is increasing. Elderly people are the fastest growing population and chronic kidney disease (CKD) is a substantial concern in this population. With aging, various structural changes take place in the kidneys, including the occurrence of renal cysts, cortical thinning, and nephrosclerosis (two or more of arteriosclerosis, focal glomerulosclerosis, tubular atrophy, and interstitial fibrosis). Moreover, kidney function decreases with age, and this decrease is correlated with structural changes [1].

Periostin is a matricellular protein that promotes tissue regeneration, fibrosis, and wound healing. It is involved in the normal development of teeth, bones, the heart, and the kidneys during embryonic development, but is not detected in adults [2, 3]. However, periostin expression has been associated with various pathological conditions, such as asthma [4], heart failure [5], myocardial infarction [6], and metastasis of various cancers [7].

Research on periostin is emerging in the field of nephrology. Periostin is mainly expressed in tubulointerstitial areas in renal fibrosis, and urinary periostin levels have been shown to be related with tubular damage [8]. Recent studies have reported the importance of periostin as a tissue or urinary biomarker in type 2 diabetes [9], lupus nephritis [10], and IgA nephropathy [11]. We previously reported that inhibition of periostin led to a decrease in renal inflammation and fibrosis *in vivo* and *in vitro* [12, 13]. Fibrosis is a common pathway after kidney damage and an important mechanism of kidney aging. Changes in lipid metabolism during kidney aging have been recently reported [14].

In the current study, we used wild-type (WT) B6 mice and periostin *(Postn)-null* mice of 2 and 24 months of age to investigate the role of periostin in kidney aging and to elucidate whether changes in lipid metabolism are involved. Lipid species were identified and quantitatively analyzed by the multiple reaction monitoring (MRM) method using a linear ion trap triple quadrupole mass spectrometry (MS) instrument. MS-based platforms are widely used for targeted lipidomics because of their excellent resolution, high sensitivity and selectivity, and wide dynamic range [15, 16]. MRM-based analysis allows quantifying various lipids simultaneously by detecting accurate precursors and product ions of low-concentration molecules in a sample [17]. Quantitative analysis of selected lipids using the MRM method is fast and simple as it bypasses lipid identification.

To predict homeostasis disorders or health conditions, it is important to study the pathways and networks of biological metabolites affected by or involved in aging. Metabolic profiling to identify and quantify aging-related lipids using MS has been reported in various studies [18–21]. However, the link between periostin-induced aging and lipids has not been identified to date. We investigated changes in the kidneys due to aging and the effects of periostin ablation thereon. In addition, we elucidated the changes in the renal lipid profile according to aging and periostin expression.

## RESULTS

### Gross appearance and renal function in young and aged WT and *Postn-null* mice

**Figure 1:**
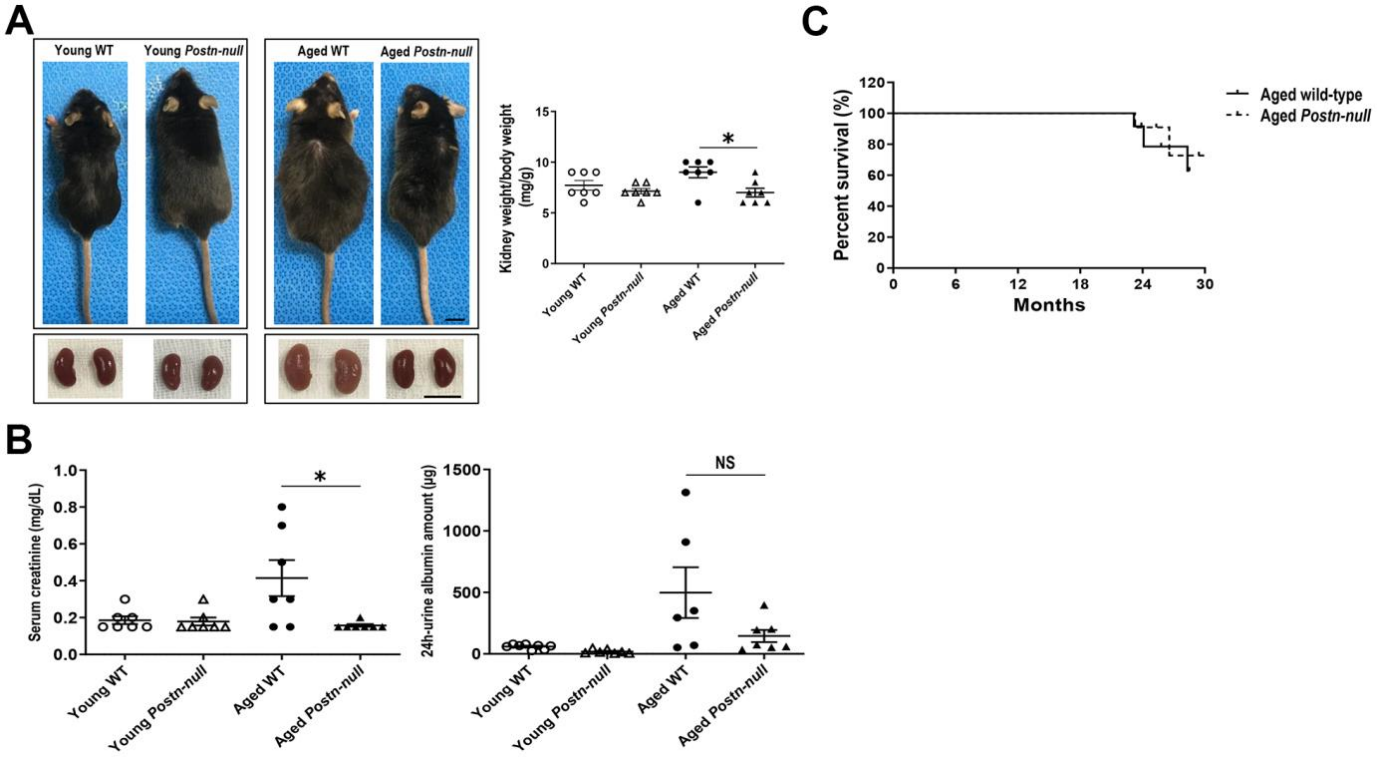
**Gross appearance and renal function in young and aged WT and *Postn-null* mice.** (**A**) Aged *Postn-null* mice had smaller body and kidney sizes than aged WT mice. Representative data are shown (N = 7/group) (bar: 1 cm). Data are the mean ± SEM. *p < 0.05 (unpaired *t*-test). (**B**) Albuminuria and serum creatinine were reduced in aged *Postn-null* mice. Data are the mean ± SEM. *p < 0.05 (unpaired *t*-test). (**C**) There was no difference in the survival rate between aged *Postn-null* mice and aged WT mice.

Aged WT mice were larger than young WT mice and had a higher kidney weight-to-body weight ratio. In *Postn-null* mice, there were no significant differences in size and kidney weight-to-body weight ratio between aged and young mice. In addition, aged *Postn-null* mice were smaller and had a lower kidney weight-to-body weight ratio than aged WT mice (Figure 1A). Serum creatinine levels were higher in aged WT mice than in aged *Postn-null* mice. Albuminuria tended to be lower in aged *Postn-null* mice, but the difference was not statistically significant (Figure 1B). There was no significant difference in the survival rate between *Postn-null* mice and WT mice (Figure 1C).

### Role of periostin in renal fibrosis due to aging

**Figure 2:**
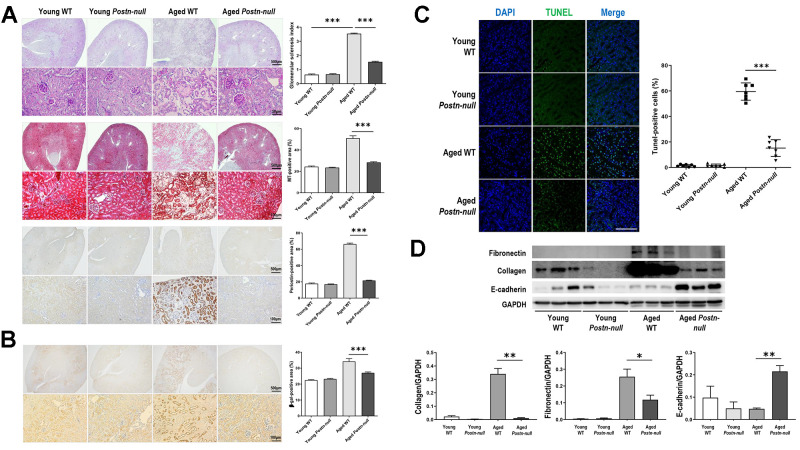
**Role of periostin in renal fibrosis due to aging.** (**A**) Tubular atrophy, interstitial fibrosis, glomerular sclerosis, and periostin expression were increased in aged WT mice, but not so much in aged *Postn-null* mice. Representative data are shown (N = 7/group). Magnification: 40× (top); 400× (bottom); 200× (bottom). Data are the mean ± SEM. ***p < 0.001 (unpaired *t*-test). (**B**) Beta-galactosidase expression was increased in aged WT mice, but a lesser extent in aged *Postn-null* mice. Representative data are shown (N = 7/group). Magnification: 40× (top); 200× (bottom). Data are the mean ± SEM. ***p < 0.001 (unpaired *t*-test). (**C**) Apoptotic cells were significantly increased in aged WT, but to a markedly lower level in aged *Postn-null* mice. Data are the mean ± SEM. ***p < 0.001 (unpaired *t*-test). (**D**) The expression of fibrosis markers was increased in aged WT mice, but not so much in aged *Postn-null* mice. Data are the mean ± SEM. *p < 0.05; **p < 0.01; ***p < 0.001 (unpaired *t*-test).

Tubular atrophy/interstitial fibrosis was more prominent in aged WT mice than in young WT mice and aged *Postn-null* mice (Figure 2A). The glomerular sclerosis index increased in aged WT mice, but to a lesser extent in aged *Postn-null* mice. Periostin was more strongly expressed in the tubular areas in aged WT mice than in young WT mice. Senescence-associated beta-galactosidase was more strongly expressed in aged WT mice than in young WT mice, while its expression was significantly lower in aged *Postn-null* mice (Figure 2B). TUNEL staining revealed that apoptotic cells were significantly increased in aged WT mice, but to a markedly lower level in aged *Postn-null* mice (Figure 2C). The expression levels of fibronectin and collagen, which were increased in aged WT mice compared to young WT mice, were attenuated in aged *Postn-null* mice (Figure 2D). In contrast, periostin depletion led to a significant increase in E-cadherin expression when compared with that in aged WT mice.

### Lipid profiles according to kidney age and periostin expression

**Figure 3:**
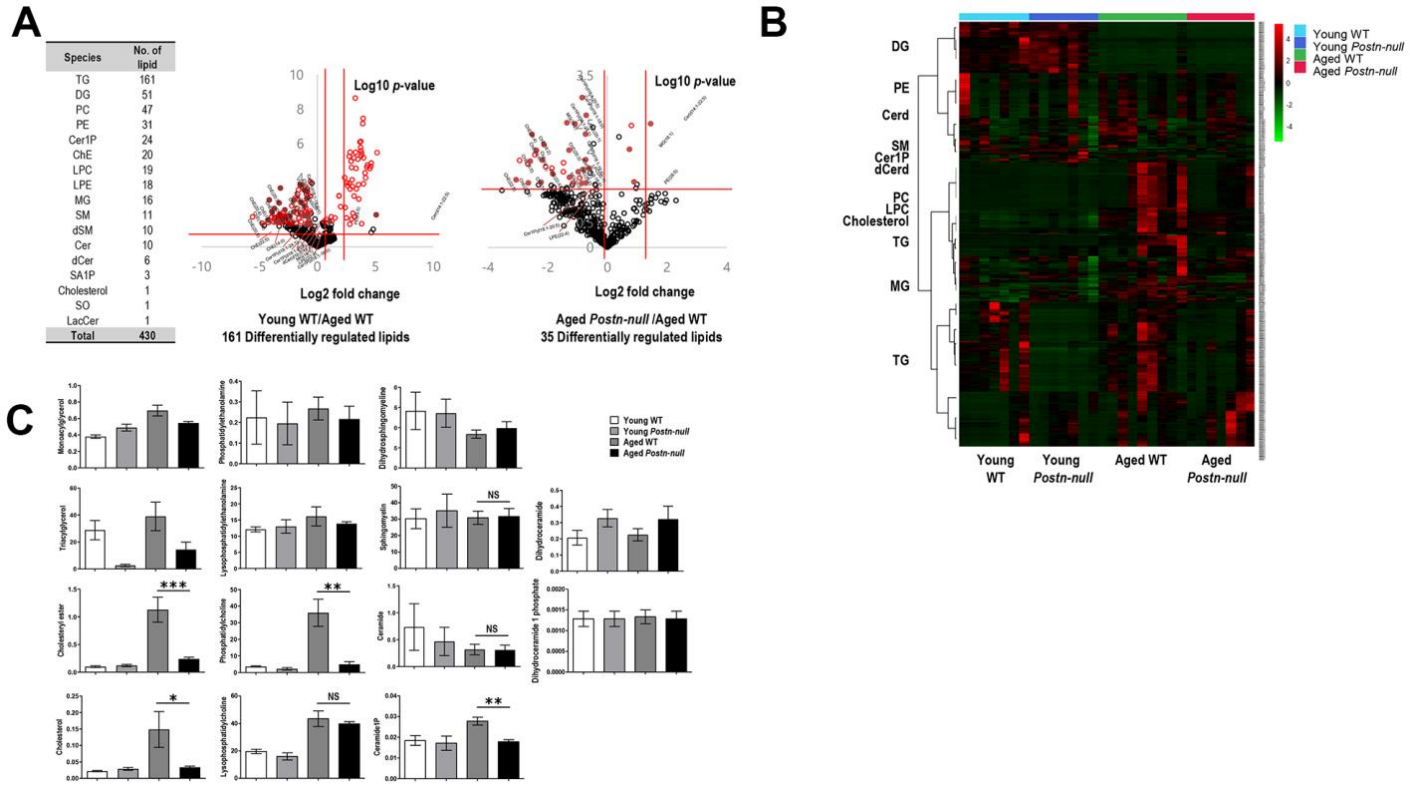
**Overall lipid profiles according to kidney age and periostin expression.** (**A**) Numbers of lipids identified. In total, 430 lipid species of 17 classes were identified. The volcano plot shows the magnitude and significance of the fold changes in young WT mice group (middle) and aged *Postn-null* mice (right) versus aged WT mice. The two vertical red lines indicate the |1.5|-fold change, and the horizontal line indicates a *p*-value of 0.05. Red dots are DRLs. (**B**) Clustering heatmap of the 430 lipids identified. (**C**) Lipid levels per class in the four study groups. Data are the mean ± SEM. Statistical significance was evaluated using Tukey tests. *p < 0.05; **p < 0.01; ***p < 0.001.

We performed lipidomic analysis of kidney samples from aged WT, aged *Postn-null*, young WT, and young *Postn-null* mice. Targeted lipids identified using the MRM mode were quantified following normalization to an internal standard (IS) for each lipid class. In total, 430 lipid species of 17 lipid classes were detected (Figure 3A). Using the quantitative lipid values, fold changes and *p*-values (Student’s *t*-test) were calculated for individual lipid species. Volcano plots were drawn to visualize the statistical significance and the magnitude of the fold change of differentially regulated lipids (DRLs) in aged WT mice versus young WT mice and in aged *Postn-null* mice versus aged WT mice (Figure 3A).

DRLs were identified based on a fold change > 1.5 or < 0.67, and *p* < 0.05. In total, 161 and 35 DRLs were identified in young WT mice versus aged WT mice and aged *Postn-null* mice versus aged WT mice, respectively (Supplementary Table 1). Twelve cholesteryl esters (chEs), four monoacylglycerols (MGs), four ceramide-1-phosphates (C1Ps), and one ceramide (Cer), dihydroceramide (dCer), lysophosphatidylcholine (LPC), lysophosphatidylethanolamine (LPE), and phosphatidylethanolamine (PE) were selected. The expression of all 12 chEs was elevated in aged WT mice. Figure 3B shows a heatmap of the expression levels (z-scores) of the 430 lipids identified in the four groups. There were clear differences between aged WT mice and aged *Postn-null* mice.

### Changes in each lipid class according to kidney age and periostin expression

**Figure 4:**
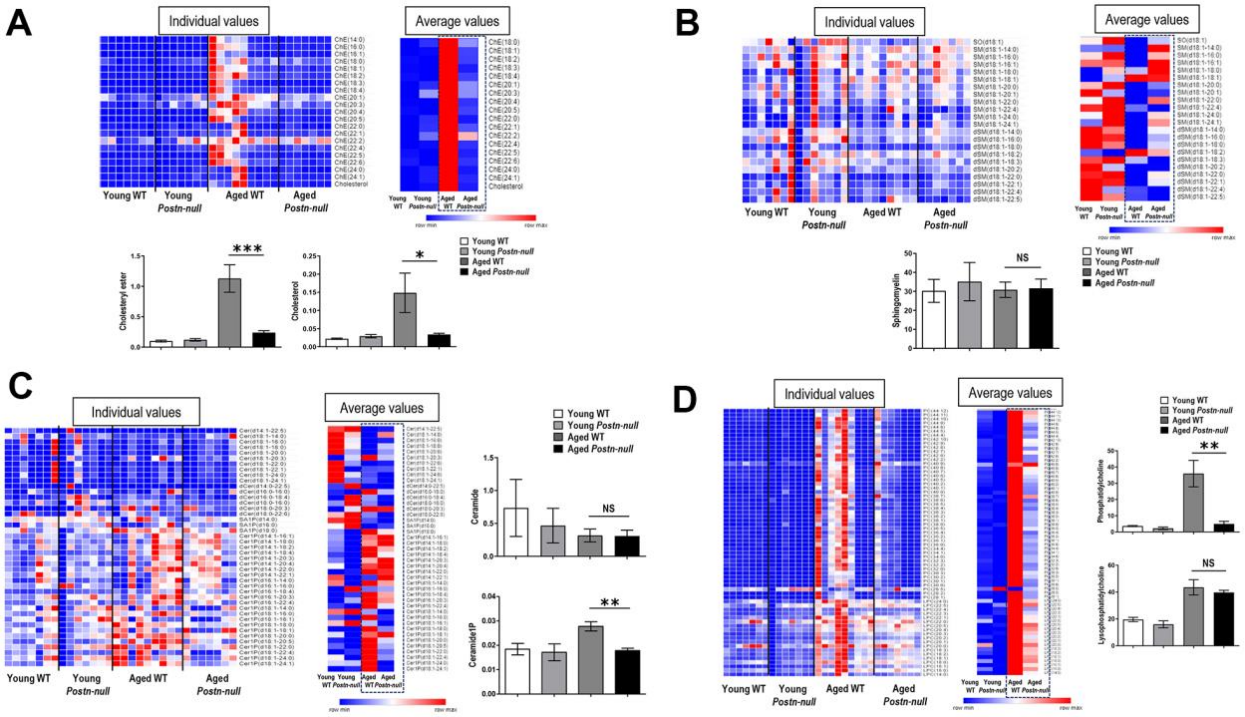
**Changes in lipid profiles according to kidney age and periostin expression.** (**A**) chE and cholesterol were significantly increased in aged WT mice compared to young mice, but to a lesser extent in aged *Postn-null* mice. Data are the mean ± SEM. *p < 0.05 (unpaired *t*-test); ***p < 0.001 (unpaired *t*-test). (**B, C**) SM and Cer did not differ between the groups, but C1P was higher in aged WT mice than in aged *Postn-null* mice. Data are the mean ± SEM. **p < 0.01 (unpaired *t*-test). (**D**) LPC was increased in all aged mice, with no difference between WT mice and *Postn-null* mice. In contrast, PC was significantly lower in aged *Postn-null* mice than in aged WT mice. Data are the mean ± SEM. **p < 0.01 (unpaired *t*-test).

To confirm the change in the level of each lipid class according to age and periostin expression, heatmaps were drawn using z-scores to visualize lipid expression in individual samples of each group and the average expression in the group and relative expression levels of lipids in each group were represented as bar graphs (Figure 3C). The chE and cholesterol levels were higher in aged WT mice than in the other groups (Figure 4A). Their levels were significantly lower in aged *Postn-null* mice than in aged WT mice. The relative abundances of polyunsaturated fatty acids (PUFAs) in the four groups are shown in Supplementary Figure 1. Among them, linoleic acid (18:2), linolenic acid (20:3), arachidonic acid (20:4), and docosahexaenoic acid (22:6) showed the largest differences between aged WT and aged *Postn-null* mice. Except for linolenic acid, PUFA levels were the highest in aged WT mice.

The levels of sphingosine (SO), dihydrosphingomyelin (dSM), and sphingomyelin (SM) were the lowest in aged WT mice (Figure 4B). There was little difference in average SM lipid class expression between aged WT mice and aged *Postn-null* mice. Cer expression was lower in aged WT mice than in young WT mice and did not significantly differ between aged WT mice and aged *Postn-null* mice (Figure 4C). C1P expression was the highest in aged WT mice and was significantly lower in aged *Postn-null* mice than in aged WT mice. The expression of CCL5 was also significantly different between the two groups (Supplementary Figure 2).

The levels of phosphatidylcholine (PC) and LPC were the highest in aged WT mice (Figure 4D). LPC was increased in all aged mice, and there was no difference between aged WT mice and aged *Postn-null* mice. In contrast, PC was significantly increased in aged WT mice when compared to aged *Postn-null* mice. Furthermore, RNA sequencing and real-time reverse transcription (RT-q) PCR revealed that LPC acyltransferase (LPCAT2), which converts LPC to PC, was increased in aged WT mice, but not in aged *Postn-null* mice (Supplementary Figure 3). The levels of MG, PE, LPE, and triacylglycerol (TG) were lower in aged *Postn-null* mice than in aged WT mice, but the difference was not significant (Figure 3C).

### Changes in lipid metabolism according to kidney age and periostin expression

**Figure 5:**
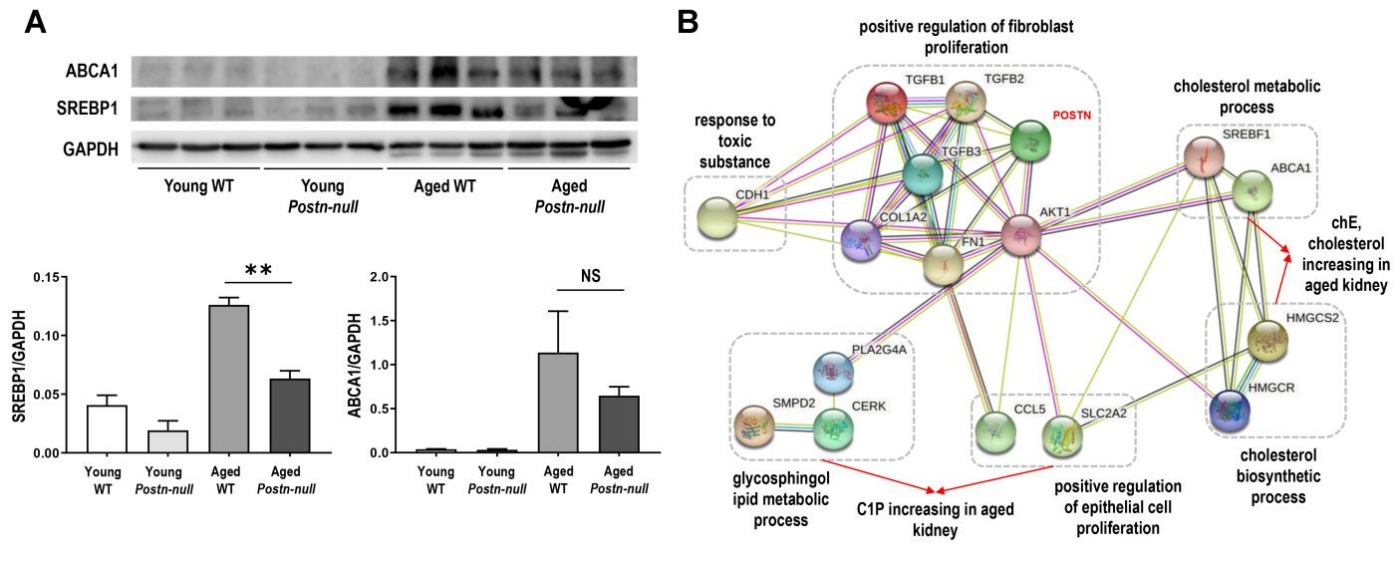
(**A**) SREBP1 and ABCA1 protein expression levels. Data are the mean ± SEM. **p < 0.01 (unpaired *t*-test). (**B**) Protein-protein interaction network of periostin and important factors in aging. Pathways are indicated by dotted lines based on clustering of the proteins according to their biological processes using STRING. ABCA1, ATP binding cassette transporter 1; AKT1, AKT Serine/Threonine Kinase 1; CCL5, C-C Motif Chemokine Ligand 5; CDH1, Cadherin 1; CERK, Ceramide Kinase; COL1A2, Collagen Type I Alpha 2 Chain; FN1, Fibronectin 1; HMGCR, 3-Hydroxy-3-Methylglutaryl-CoA Reductase; HMGCS2, 3-hydroxy-3-methylglutaryl-CoA synthase 2; PLA2G4A, Phospholipase A2 Group IVA; POSTN, Periostin; SLC2A2, Solute Carrier Family 2 Member 2; SMPD2, Sphingomyelin Phosphodiesterase 2; SREBF1, Sterol Regulatory Element Binding Transcription Factor 1; TGF, Transforming growth factor.

We investigated the protein expression of sterol regulatory element-binding protein (SREBP1) and ATP-binding cassette transporter A1 (ABCA1), which are involved in lipid metabolism, in the study samples (Figure 5A). The expression of both SREBP1 and ABCA1 was increased in aged WT mice and decreased in aged *Postn-null* mice when compared to that in young WT mice.

### Construction of an aging-related protein-protein interaction network

A network was established to systematically understand the interactions among, and biological functions of proteins enriched with aging (Figure 5B). When the proteins were clustered according to biological process and mechanisms related to fibroblast proliferation, cholesterol and glycosphingolipid metabolic processes were upregulated with aging. The network analysis confirmed that proteins involved in chE and cholesterol biosynthetic processes, which are known to induce fibrosis, were enriched with aging. In addition, glycosphingolipid metabolism was upregulated, which leads to increases in Cer, C1P, and SM lipids. Cer, C1P, and SM are known to be closely related to apoptosis, cell proliferation, and inflammation mechanisms. This network showed that various processes, such as fibrosis and lipid metabolism, are more activated with aging.

## DISCUSSION

We demonstrated that periostin expression in the kidneys increased with age in mice. Decreased renal function, tubular atrophy/interstitial fibrosis, glomerular sclerosis, and increased fibrotic marker expression and apoptosis in aged WT mice were improved or attenuated in aged *Postn-null* mice. In addition, lipid profiling data indicated that chE, cholesterol, C1P, and PC were significantly increased in aged WT mice, whereas their levels were lower in aged *Postn-null* mice.

Several mechanisms are involved in kidney aging: fibrosis-related mechanisms, such as increases in extracellular matrix production and tissue inhibitors of metalloproteinases, Wnt signaling, and vascular calcification, and vascular mechanisms, such as intimal thickening, inflammation-related mechanisms, increased oxidative damage, and increased cell senescence. These processes are accompanied by structural and functional changes [1, 22].

Periostin is associated with various mechanisms, including fibrosis, inflammation, and apoptosis, and is strongly associated with the occurrence and progression of kidney aging and the resulting deterioration of renal function. In patients with IgA nephropathy, urine periostin levels were highly correlated with renal histological findings and were closely related to long-term kidney prognosis [11]. In animal and cell models of CKD or acute kidney injury-to-CKD transition, periostin and disease severity were significantly related, and renal injury was alleviated upon inhibition of periostin [13, 23]. Similar findings have been reported in *Postn-null* mice [12]. Based on the above experimental results, periostin ablation was expected to have an inhibitory and protective effect on renal aging. In fact, although we did not use a disease model, our findings demonstrated that periostin expression increased with the deterioration of renal function and histological changes in aged WT mice, whereas *Postn-null* mice did not show such functional or histological changes.

Aging-related changes in lipids have been reported in various tissues and fluids, including serum [19, 24], brain [20], liver, kidneys [18], heart [25], and mitochondria [26]. However, due to the natural variability of biological samples according to sex [19], study region [27], and aging period [28], the changes in lipids during aging may differ among studies. Recently, changes in PC, PE, SM, phosphatidylserine (PS), and Cer in aged mice have been reported [14]. An increase in ceramide hydrolase decreases ceramide C16, resulting in changes in cell survival and apoptosis, and cytoskeletal rearrangements. The catabolic product itself is involved in renal fibrosis; thus, it was found that ceramide metabolism directly affects the aging process.

However, the results of our study differed slightly from the above results. First, the expression of cholesterol and chE was significantly increased in aged WT mice, but not so much in aged *Postn-null* mice. Among them, PUFAs, including linoleic acid, linolenic acid, arachidonic acid, and docosahexaenoic acid, showed significant differences. PUFAs are known to exhibit a protective effect on brain aging [29, 30], likely by attenuating neuroinflammation, peroxidative damage, and vascular aging. It is thought that the same mechanism slows down aging in the kidneys. In fact, their levels were high in aged WT mice, but relatively low in aged *Postn-null* mice, indicating that PUFAs may be increased in a protective/compensatory response to aging, rather than being a cause of aging.

Second, SM and Cer levels did not differ between aged *Postn-null* mice and aged WT mice, whereas C1P was significantly lower in aged *Postn-null* mice than in aged WT mice, and increased C1P regulated CCL5 expression as indicated by RNA sequencing and mRNA expression analysis. Increased C1P or CCL5 is considered to affect kidney aging and fibrosis by participating in inflammation, cell invasion/migration, and cell survival/proliferation [31–35]*.* The association between C1P, glomerular damage, and proteinuria has been previously reported [36]. Moreover, it is associated with inflammation in obesity, diabetes, autoimmune diseases, and cancer [37–39]. C1P stimulates cell survival and prevents apoptosis, causing excessive cell proliferation and exerting toxic effects [40, 41]*.* Further, it can exert a proinflammatory effect [42, 43]*.* CCL5 is expressed in many cells, including kidney epithelial and endothelial cells. It is known to recruit inflammatory cells to damaged tissues, induce kidney damage through RAS activation, increase blood pressure, and cause renal fibrosis [44–46]. In line with the contradictory roles of C1P and CCL5 reported in previous studies, in our study, aging and fibrosis may have been induced by C1P and CCL5.

Finally, there was no difference in LPC, but the PC level and LPCAT2 was significantly higher in aged WT mice than in young mice or aged *Postn-null* mice. LPC has harmful effects, including destruction of mitochondrial integrity, increased oxidative stress, increased expression of inflammatory cytokines, inhibition of cell proliferation, and apoptosis [47]. LPCAT2 is important for maintaining LPC levels and thus, homeostasis. In addition, LPCAT2 regulates the inflammatory response in innate immune cells [48] and decreases the effectiveness of anticancer drugs [49]. Its expression is increased in various cancers [50]. Therefore, it was thought that the expression of LPCAT2 increased because of aging in aged WT mice, that is, as a compensatory effect, and this resulted in an increase in PC levels.

Based on the results of this study alone, we were not able to directly correlate and interpret changes in periostin and lipids. Therefore, we investigated the levels of SREBP1 and ABCA1, which are involved in lipid metabolism. Our results were consistent with the finding in a previous study that an increase in SREBP1 expression in the kidneys with aging induces glomerulosclerosis and proteinuria, leading to nephropathy due to aging [51]. Moreover, SREBP1 may be important in renal fibrosis progression by having a direct role in matrix regulation in addition to the regulation of lipid homeostasis [52]. Downregulation of ABCA1, responsible for the efflux of intracellular free cholesterol and phospholipids through the plasma membrane, is known to play an important role in the occurrence of various diseases by causing pathologic atherogenesis through disrupting cholesterol removal from the cytoplasm [53]. Our results suggest that the increase in ABCA1 may have been a compensatory response to the lipid profile changes caused by aging rather than a cause of kidney aging. In line herewith, Jiang et al. have reported that *ABCA1* mRNA expression and SREBP-1 expression were higher in 23-month-old C57BL/6 mice than in young mice [51].

As the aging phenomenon is not consistent among individuals, several results varied even within the same group. Serum creatinine showed significant differences between the two groups (aged WT vs. aged *Postn-null*), but there were also within-group differences. Albuminuria was increased in aged WT mice and substantially less so in aged *Postn-null* mice, but the difference was not statistically significant. To confirm the differences more clearly, future studies using larger study populations will be needed.

Nevertheless, the results of this study demonstrated that periostin ablation delays aging and that periostin inhibition is associated with changes in several lipids and lipid metabolism. Further research on the precise mechanisms of and relationships between lipid expression and metabolism, kidney aging, and periostin expression is warranted.

## MATERIALS AND METHODS

### Experimental animals

All experiments were performed in accordance with the Guidelines for the Care and Use of Laboratory Animals of the National Research Council and the National Institutes of Health with the approval of the Institutional Animal Care and Use Committee of the Clinical Research Institute at Seoul National University Boramae Medical Center (no. 2018-0049). Male WT mice (C57BL/6) (Koatech, Seoul, Korea) and male *Postn-null* mice [54] (C57BL/6; 129-Postn^tm1Jmol^/J; the Jackson Laboratory, Bar Harbor, ME, USA) were raised in a specific pathogen-free animal facility at 22 ± 2° C with 40–60% humidity, air pressure of 5 mmH_2_O, illuminance of 150–300 lx, noise of 60 dB or less, and ventilation was performed 10–20 times per hour. To evaluate the role of periostin in lipid metabolism during kidney aging, we randomly divided male WT and *Postn-null* mice into four groups, comprising young (2-month model) and aged (24-month model) groups. Survival analysis was conducted.

### Blood and urine chemistry data

Two- and 24-month model mice were sacrificed and sampled per to the same protocols. Before sampling, urine was collected during 24 h from mice that were observed in a metabolic cage. One day later, blood and tissue samples, including the kidneys, were collected after anesthesia with xylazine (Rompun; 10 mg/kg; Bayer, Canada) and Zoletil™ (30 mg/kg; Virbac, Fort Worth, TX, USA). The blood samples were immediately analyzed using an i-STAT handheld blood analyzer system (Abbot Point of Care, Princeton, NJ, USA). Urine albumin levels (μg/mL; Albuwell M, Exocell, Newtown Square, PA, USA) were measured using the 24-h urine samples. The amount of urine albumin (μg) was calculated by multiplying the urine albumin concentration by the amount of urine for 24 h.

### Histological analyses

Four-micrometer-thick paraffin sections were stained with periodic acid–Schiff reagent and Masson’s trichrome and observed under a light microscope. Tubular atrophy and interstitial fibrosis were scored from 0 to 5 by a renal pathologist who was blinded to the experimental groups. At least 10 fields (at a magnification of 200×) per section were evaluated for each sample. A minimum of 50 glomeruli per mouse kidney were evaluated, and the mean value is reported for each mouse. The glomerular sclerosis index was semi-quantitatively scored from 0 to 4 (0, normal; 1, mesangial thickening of *<* 25% of the tuft; 2, mesangial proliferation and thickening up to 50%; 3, obliteration of capillaries and diffuse sclerosis up to 75%; and 4, complete capillary obliteration and thrombosis with global sclerosis up to 100%) [55, 56].

For immunohistochemical assays, sections were deparaffinized and hydrated using xylene and ethanol. Endogenous streptavidin activity was blocked with 3% hydrogen peroxide. The deparaffinized sections were stained with an anti-periostin antibody (Abcam, Cambridge, MA, USA) and then incubated with horseradish peroxidase-conjugated goat anti-rabbit IgG (Vector Laboratories, Burlingame, CA, USA). 3,3'-Diaminobenzidine tetrahydrochloride (Sigma-Aldrich, St. Louis, MO, USA) was used for immunohistochemical detection. All sections were counterstained with Mayer’s hematoxylin (Sigma-Aldrich) and evaluated under a light microscope with a differential interference contrast optics camera (Leica DFC-295, Wetzlar, Germany). For each sample, at least 10 fields (magnification, 100×) were randomly selected, and interstitial fibrosis (Masson’s trichrome-positive) and periostin-positive areas were quantified by computer-assisted morphometry (Qwin 3, Leica, Mannheim, Germany). Scores were determined in a blinded manner using the mean values of the positive areas (%).

### Beta-galactosidase staining

Senescence-associated β-galactosidase staining was performed using a Senescence β-Galactosidase Staining Kit (Cell Signaling Technology, Danvers, MA, USA) according to the manufacturer’s protocol.

### TUNEL staining

Apoptotic cells were detected using an ApopTag Fluorescein *In Situ* Apoptosis Detection kit (Roche, Mannheim, Germany). Paraffin-embedded kidney slides were deparaffinized, hydrated, and sequentially incubated with protease K (Dako, Santa Clara, CA, USA), terminal deoxynucleotidyl transferase enzyme, and anti-digoxigenin-fluorescein (Roche). The slides were mounted with Gold antifade reagent containing 4',6-diamidino-2-phenylindole (Invitrogen, Carlsbad, Calif, USA) and examined under a fluorescence microscope. For each sample, five fields (400×) were randomly selected, and the staining was quantified using computer-based morphometric analysis (Qwin 3, Leica, Mannheim, Germany). Scoring was performed in a blinded manner using the mean values of the positive areas (%).

### RT-qPCR

Briefly, 1 μg of total RNA extracted from mouse kidney tissues using the RNeasy Kit (Qiagen, Hilden, Germany) was reverse-transcribed using oligo-d(T) primers and AMV-RT Taq polymerase (Promega, Madison, WI, USA). qPCRs were run using Assay-on-Demand TaqMan probes and primers targeting *LPCAT2* and glyceraldehyde 3-phosphate dehydrogenase (*GAPDH*; Applied Biosystems, Foster City, CA, USA) on an ABI PRISM 7500 Sequence Detection System (Applied Biosystems). After normalization to *GAPDH* expression, mRNA levels were calculated using the comparative Ct method (2^–ΔΔCt^).

### RNA sequencing

Total RNA was extracted using RNeasy Mini kits (Qiagen, Hilden, Germany) according to the manufacturer’s instructions. The RNA was stored at –80° C, and RNA sequencing and read alignments were conducted at ChunLab (Seoul, Korea). RNA quantity and quality were evaluated using an Epoch™ Spectrometer (BioTek, Winooski, VT, USA) and 2100 Bioanalyzer (Agilent Technologies, Santa Clara, CA, USA). All samples met the following quality criteria: amount ≥1.1 μg, concentration ≥50 ng/μL, volume ≥20 μL, A260/280 ≥1.8, RNA integrity number ≥7, rRNA ratio (23S/16S or 28S/18S) ≥1.3.

### Western blot analysis

Proteins were extracted from homogenized kidney tissues using RIPA buffer (Biosesang, Seongnam, Korea) and protein concentrations were determined using the BCA assay (Thermo Fisher Scientific, Waltham, MA, USA). Equal amounts of proteins were electrophoresed with glycine-sodium dodecyl sulfate buffer (LPS Solution, Daejeon, Korea) and transferred to polyvinylidene difluoride membranes (Millipore, Bedford, MA, USA) on ice. After the membranes were blocked with 5% skim milk (Becton Dickinson Rowa France, Le Pont-de-Claix, France) containing 2% bovine serum albumin, they were incubated overnight at 4° C under shaking with primary antibodies against E-cadherin (Abcam, Cambridge, UK), fibronectin, collagen, ABCA1, SREBP1, and GAPDH (Cell Signaling Technology). After 24 h, the membranes were incubated with a mouse or rabbit IgG-conjugated secondary antibody (Cell Signaling Technology) for 1 h. The protein bands were observed using the enhanced chemiluminescence method (Advansta, San Jose, CA, USA) and were quantified by densitometry using in ImageJ (National Institutes of Health, Bethesda, MD, USA).

### Protein-protein interaction network construction

We built a protein-protein interaction network to identify the interactions among and biological processes of proteins in the kidneys that are upregulated with aging. Gene Ontology biological processes of proteins that had been validated as important factors in aging by western blot and RT-qPCR analyses were analyzed using the STRING software [57]. Using this software, the network was systematically built to understand the interactions between proteins that are physically or functionally related. In the network, each node represents a protein, and the lines connecting the nodes indicate physical or functional relationships, and the functional organization is indicated by function clusters indicated with dotted lines.

### Lipid standards

Lipid standards [MG (15:1), diacylglycerols (8:0-8:0), TG (11:1-11:1-11:1) chE (10:0)] were purchased from Larodan Fine Chemicals AB (MI, USA). Other lipids [Cer (d18:1-12:0), dCer (d18:0-12:0), C1P (d18:1-12:0), dihydroceramide-1-phosphate (d18:0-16:0), SO (17:1), sphinganine; SA (17:0), SO-1-phosphate (17:1), sphinganine-1-phosphate (17:0), SM (d18:1-12:0), dSM (d18:1-12:0), PC (10:1-10:1), LPC (13:0), PE (10:1-10:1), LPE (14:0), PG (10:1-10:1), LPG (14:0), phosphatidic acid (10:1-10:1), lysophosphatidic acid; LPA (17:0), PS (10:1-10:1), lysophosphatidylserine (17:1), phosphatidylinositol (16:0), lysophosphatidylinositol (13:0)] were purchased from Avanti Polar Lipids, Inc (AL, USA). ChE was dissolved in chloroform and all other lipid standards were dissolved in methanol, stored at –20° C, and diluted to 1 μg/mL for use in lipid extraction of the sample.

### Lipid extraction

A two-step lipid extraction method [58] were applied by combining the Folch method and the Bligh and Dyer method to obtain glycolipids, cholesterol, cholesteryl ester, glycophospholipids, and sphingolipids. Twenty milligrams of pulverized whole kidney tissue were added to 660 μL of methanol, 330 μL of chloroform, and 1 μg/mL of a mixed lipid IS. The samples were incubated on ice for 10 min, vortexed for 30 s every 3 min, and then centrifuged at 13,800 × *g*, 4° C for 2 min. The first extracted lipid solvent, the supernatant, was transferred to a new tube, and the pellet was added to 496 μL of methanol, 248 μL of chloroform, and 6 μL of 37% HCl. The samples were incubated on ice for 15 min and vortexed every 5 min for 30 s, then 250 μL of cold chloroform and 450 μL of 0.1 N HCl were added, and the samples were vortexed for 1 min and centrifuged at 6,500 × *g*, 4° C for 2 min. The organic phase, i.e., the bottom layer, was collected, mixed with the first extracted lipid solution. The mixture was divided into two equal aliquots into and dried with a SpeedVac concentrator. One aliquot was dissolved in 100 μL of solvent mixture (sol A:sol B, 2:1, v/v) to analyze positive and neutral lipid ions, and the other was dissolved in 100 μL of methanol for trimethylsilyldiazomethane (TMSD) methylation to analyze anionic lipids [59]. For TMSD methylation, an equal volume of 2 M TMSD in hexane was mixed in the sample. Then, the sample was vortexed for 30 s and incubated at 37° C for 15 min. To quench the reaction, acetic acid was added at5% of the total volume of. Finally, the samples were subjected to LC-MS.

### Quantitative lipid analysis using LC-MS

An Acquity UPLC system (Waters, Milford, MA, USA) equipped with a Hypersil GOLD column (2.1 × 100 mm; 1.9 μm, Thermo Fisher Scientific) was used to separate lipids according to hydrophobicity. The column was maintained at 40° C and the sample tray at 4° C, and the flow rate was set to 0.3 ml/min. Solvent A (methanol:acetonitrile:water, 19:19:2, v/v/v, 20 mM ammonium formate, 0.1% (v/v) formic acid) and solvent B (isopropanol, 20 mM ammonium formate, 0.1% (v/v) formic acid) were prepared and a 33-min gradient elution method was set up as follows: solvent B, 5%; 0–5 min, 30%; 5–15 min, 90%; 15–22 min, 90%; 22–25 min, 5%; 25–26 min, and 5%, 26–33 min. Chemical derivatization of polar lipids reduces peak tailing and shows high stability and ionization efficiency in LC-MS, and low abundance lipids can also be analyzed [60]. QTRAP 5500 (AB Sciex, Foster City, CA, USA) is a linear ion trap MS instrument with a triple quadrupole, and parameters were set up as follows: curtain gas: 20, collision gas: high, ion spray voltage: positive mode of 5500 V, temperature: 300–500° C, ion source gas 1: 30–60 psi, ion source gas 2: 60–85 psi. Ultra-high-purity nitrogen was supplied for collision. Multiple-reaction monitoring (MRM) is a highly specific and sensitive targeted ion quantification method that can selectively analyze precursor ions and daughter ions of fragmented with tandem MS. The QTRAP 5500 (AB Sciex) MS instrument has a triple quadrupole, and the first quadrupole (Q1) transmits only precursor ions with a specific m/z. Then, in the second quadrupole (Q2), the ions were fragmented, and a specific product ion of m/z is selected and moved in the third quadrupole (Q3). By selecting the analyte with high sensitivity through ion selection processes of the MRM method, it is possible to effectively analyze and quantify complexity of lipids.

### Lipid data processing

Lipids were analyzed using the MRM method according to previously optimized conditions [61]. The MRM scan mode was set up as follows: MRM transition setting depending on lipid class; entrance potential, 10; collision cell exit potential, 14. MRM data were acquired by LC-MS using the Analyst MassHunter Workstation Data Acquisition software (AB Sciex, Foster City, CA, USA). MRM data of target lipids assigned to the set precursor ion and product ion were exported using Qualitative Analysis software, version B.06.00 (Agilent Technologies, DE, USA). Using Skyline software (MacCoss Laboratory, University of Washington, WA, USA), the peaks of lipid species were assigned and normalized compared to the retention time of the ISs of the respective lipid classes. For retention time values that vary according to the lipid species, refer to the LIPID MAPS Lipidomics Gateway (https://www.lipidmaps.org/). Heatmap cluster analysis was conducted using the MetaboAnalyst tool (https://www.metaboanalyst.ca/) [62]. Lipid levels were visualized in volcano plots based on negative log10 *p*-values (Student’s *t*-test) and log2 fold changes.

### Statistical analysis

Data are expressed as the mean ± standard deviation or median (interquartile range). Student’s *t*-test or the Mann-Whitney U test was used as relevant according to the normality of the data. For comparison of more than two groups, one- or two-way ANOVA followed by Tukey tests was used. Statistical analyses were performed using SPSS version 22 (IBM Software, Armonk, NY, USA) and GraphPad Prism 8.0 (GraphPad Software, San Diego, CA, USA). Statistical significance was set at *p <* 0.05.

## Supplementary Material

Supplementary Figures

Supplementary Table 1
