# Supplementary material for: Effects of periostin deficiency on kidney aging and lipid metabolism
Source: Aging (Albany NY). 2021 Oct 3;13(19):22649–65. doi: 10.18632/aging.203580 (PMC8544301; doi:10.18632/aging.203580)
Supplement: Supplementary Figures [file aging-13-203580-s001.pdf]

SUPPLEMENTARY FIGURES

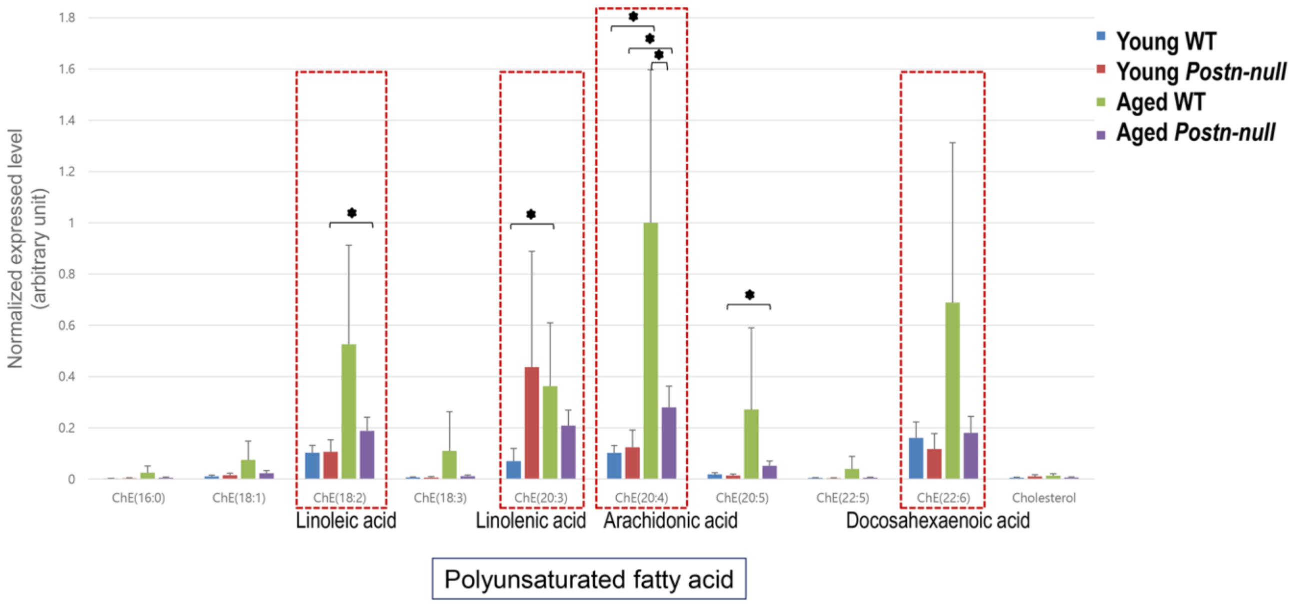

Supplementary Figure 1. Bar graph of polyunsaturated fatty acid expression in the four study groups. Arachidonic acid showed the highest expression among all polyunsaturated fatty acids (PUFA) in young WT mice, in which the expression levels was set to 1. Relative expression levels in the other groups are plotted. Data are the mean ± SEM (*p < 0.05, Tukey tests).

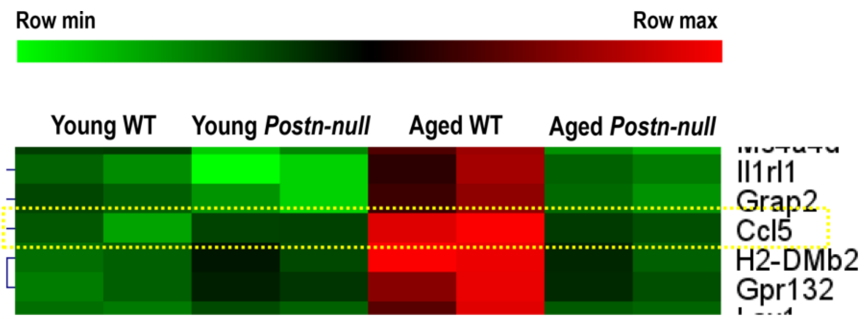

Supplementary Figure 2. CCL5 expression as assessed by RNA sequencing in the four study groups.

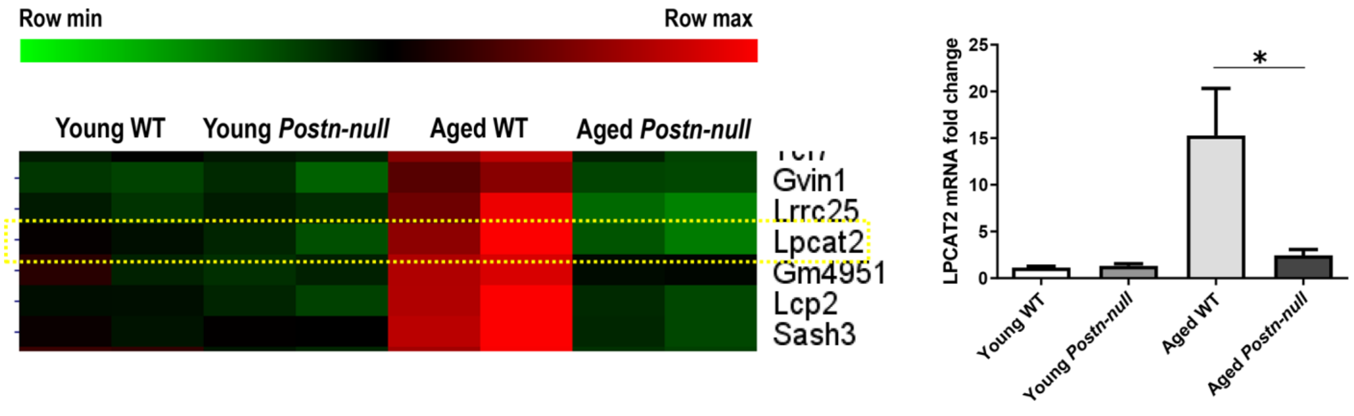

Supplementary Figure 3. LPCAT2 expression as assessed by RNA sequencing and real-time PCR in the four study groups.
