# Supplementary material for: Effects of periostin deficiency on kidney aging and lipid metabolism
Source: Aging (Albany NY). 2021 Oct 3;13(19):22649–65. doi: 10.18632/aging.203580 (PMC8544301; doi:10.18632/aging.203580)
Supplement: Supplementary Table 1 [file aging-13-203580-s002.docx]

**Supplementary Table 1. The DRL list for each group.**

**161 Differentially regulated lipids**

| **Sample** | **aged WT / young WT** | | |
| --- | --- | --- | --- |
| **No.** | **species** | **log2 FC** | **log10 p-value** |
| 1 | DG(30:5) | -5.15181 | 5.465023 |
| 2 | Cer(d14:1-22:5) | -5.08128 | 1.85215 |
| 3 | DG(40:0) | -4.64997 | 4.858044 |
| 4 | DG(42:0) | -4.60143 | 4.675219 |
| 5 | DG(38:0) | -4.52431 | 4.540633 |
| 6 | DG(36:0) | -4.32416 | 4.741083 |
| 7 | DG(44:2) | -4.26614 | 4.626882 |
| 8 | DG(32:5) | -4.15451 | 4.007395 |
| 9 | DG(44:1) | -4.10954 | 4.286123 |
| 10 | DG(30:4) | -3.98423 | 5.164506 |
| 11 | DG(44:9) | -3.98125 | 3.651859 |
| 12 | DG(30:0) | -3.95085 | 7.447877 |
| 13 | DG(28:0) | -3.908 | 7.154812 |
| 14 | DG(44:0) | -3.8303 | 5.417411 |
| 15 | DG(32:2) | -3.81875 | 1.343149 |
| 16 | DG(42:1) | -3.7693 | 5.270247 |
| 17 | DG(42:2) | -3.76049 | 5.563918 |
| 18 | DG(32:0) | -3.73926 | 6.110301 |
| 19 | DG(40:1) | -3.73402 | 5.765984 |
| 20 | DG(34:0) | -3.66864 | 6.084636 |
| 21 | DG(30:1) | -3.57374 | 2.769722 |
| 22 | DG(42:7) | -3.54927 | 3.607908 |
| 23 | DG(40:2) | -3.49474 | 5.083703 |
| 24 | DG(38:7) | -3.36949 | 3.355945 |
| 25 | DG(40:3) | -3.33258 | 4.546701 |
| 26 | DG(38:1) | -3.30188 | 5.483162 |
| 27 | DG(30:6) | -3.26787 | 8.633574 |
| 28 | DG(36:5) | -3.19879 | 1.725843 |
| 29 | DG(34:2) | -3.16707 | 2.941688 |
| 30 | DG(42:10) | -3.16277 | 5.169712 |
| 31 | DG(32:1) | -3.06403 | 1.947341 |
| 32 | DG(40:5) | -3.03379 | 3.49746 |
| 33 | DG(42:9) | -2.99394 | 3.890971 |
| 34 | DG(44:4) | -2.94813 | 4.599565 |
| 35 | DG(40:7) | -2.9458 | 1.612108 |
| 36 | DG(38:2) | -2.90105 | 4.966169 |
| 37 | DG(36:1) | -2.82453 | 5.489604 |
| 38 | DG(36:4) | -2.82225 | 3.343324 |
| 39 | DG(34:3) | -2.79954 | 1.313654 |
| 40 | DG(44:12) | -2.75 | 3.687452 |
| 41 | DG(38:6) | -2.74768 | 5.254413 |
| 42 | DG(34:1) | -2.60877 | 3.546797 |
| 43 | DG(36:2) | -2.54909 | 3.715949 |
| 44 | DG(40:8) | -2.5126 | 1.683257 |
| 45 | DG(36:3) | -2.39429 | 2.274592 |
| 46 | DG(40:4) | -2.35087 | 4.014994 |
| 47 | TG(52:6)_4 | -1.99719 | 2.329368 |
| 48 | DG(38:3) | -1.87191 | 2.949184 |
| 49 | TG(52:6)_6 | -1.8658 | 2.961265 |
| 50 | dCer(d14:0-22:5) | -1.52208 | 1.681544 |
| 51 | DG(40:6) | -1.2552 | 1.508445 |
| 52 | Cer(d18:1-14:0) | -1.198 | 1.739462 |
| 53 | PE(28:5) | -1.06205 | 1.477044 |
| 54 | dSM(d18:1-20:2) | -0.7676 | 1.590289 |
| 55 | dSM(d18:1-14:0) | -0.58085 | 1.475117 |
| 56 | MG(16:0) | 0.587278 | 3.777306 |
| 57 | MG(20:4) | 0.594631 | 2.935051 |
| 58 | MG(20:3) | 0.609021 | 2.059509 |
| 59 | Cer1P(d18:1-16:0) | 0.638891 | 1.378016 |
| 60 | Cer1P(d18:1-22:0) | 0.652998 | 1.39031 |
| 61 | LPE(22:3) | 0.703016 | 1.483847 |
| 62 | Cer1P(d14:1-22:0) | 0.72885 | 1.318357 |
| 63 | LPE(22:4) | 0.797404 | 1.383527 |
| 64 | MG(18:1) | 0.799474 | 1.725026 |
| 65 | Cer1P(d16:1-22:4) | 0.813016 | 1.33082 |
| 66 | SM(d18:1-18:1) | 0.823713 | 1.944259 |
| 67 | Cer1P(d18:1-20:0) | 0.836617 | 2.910873 |
| 68 | dCer(d16:0-18:4) | 0.854075 | 1.825783 |
| 69 | ChE(22:2) | 0.877941 | 3.227564 |
| 70 | LPC(18:0) | 0.883938 | 3.367714 |
| 71 | LPE(20:1) | 0.891183 | 1.332069 |
| 72 | MG(20:1) | 0.930962 | 2.803212 |
| 73 | LPC(16:0) | 0.940094 | 3.061113 |
| 74 | LPE(22:2) | 0.97999 | 1.992086 |
| 75 | MG(22:4) | 1.009493 | 2.282962 |
| 76 | Cer1P(d18:1-18:0) | 1.044388 | 3.289709 |
| 77 | Cer1P(d14:1-18:4) | 1.059515 | 2.062302 |
| 78 | LPC(24:0) | 1.062974 | 2.528481 |
| 79 | Cer1P(d18:1-24:1) | 1.10542 | 2.383559 |
| 80 | Cer1P(d18:1-20:5) | 1.12946 | 1.815745 |
| 81 | LPC(22:5) | 1.135234 | 3.291837 |
| 82 | LPC(18:3) | 1.212459 | 1.789227 |
| 83 | LPC(18:2) | 1.333092 | 1.942131 |
| 84 | ChE(20:1) | 1.335788 | 3.598485 |
| 85 | Cer1P(d16:1-18:4) | 1.395057 | 1.438688 |
| 86 | MG(20:2) | 1.422604 | 2.567952 |
| 87 | MG(20:5) | 1.473888 | 1.991952 |
| 88 | LPC(20:4) | 1.478856 | 3.559317 |
| 89 | LPC(22:1) | 1.486704 | 3.612723 |
| 90 | LPC(22:4) | 1.596667 | 1.677707 |
| 91 | LPC(14:0) | 1.622396 | 1.644762 |
| 92 | LPC(20:5) | 1.627129 | 3.31025 |
| 93 | LPC(20:1) | 1.708104 | 3.050309 |
| 94 | LPC(18:1) | 1.718751 | 2.768995 |
| 95 | LPC(20:2) | 1.752823 | 1.932582 |
| 96 | LPC(22:3) | 1.777183 | 2.236748 |
| 97 | PE(46:4) | 1.830591 | 1.360932 |
| 98 | LPC(20:3) | 1.936013 | 2.80969 |
| 99 | ChE(18:0) | 2.032233 | 2.703762 |
| 100 | MG(20:0) | 2.056284 | 2.023408 |
| 101 | LPC(16:1) | 2.215089 | 2.382544 |
| 102 | Cer(d18:1-20:3) | 2.273521 | 1.441589 |
| 103 | TG(56:2)_5 | 2.277062 | 1.325998 |
| 104 | PC(44:11) | 2.284605 | 1.442989 |
| 105 | PC(44:12) | 2.395939 | 1.331988 |
| 106 | ChE(22:4) | 2.398631 | 1.770101 |
| 107 | PC(40:8) | 2.648134 | 1.736334 |
| 108 | PC(44:10) | 2.681034 | 1.672547 |
| 109 | PC(40:6) | 2.682584 | 1.794218 |
| 110 | PC(42:10) | 2.762683 | 1.458125 |
| 111 | ChE(22:6) | 2.812459 | 2.140166 |
| 112 | PC(42:9) | 2.841399 | 1.544069 |
| 113 | PC(32:3) | 2.872778 | 1.721131 |
| 114 | PC(30:2) | 2.908388 | 1.501695 |
| 115 | PC(32:0) | 2.911978 | 1.655188 |
| 116 | PC(38:7) | 2.938908 | 2.013113 |
| 117 | PC(30:1) | 3.032331 | 1.450799 |
| 118 | PC(40:7) | 3.048903 | 1.818857 |
| 119 | PC(28:2) | 3.051925 | 1.453513 |
| 120 | ChE(14:0) | 3.067944 | 1.54608 |
| 121 | PC(36:5) | 3.092813 | 1.803484 |
| 122 | PC(38:2) | 3.103257 | 1.801533 |
| 123 | PC(34:1) | 3.135048 | 1.620779 |
| 124 | PC(38:5) | 3.144072 | 1.711613 |
| 125 | PC(32:2) | 3.155906 | 1.550746 |
| 126 | ChE(18:4) | 3.157814 | 1.471738 |
| 127 | PC(38:6) | 3.162446 | 1.439273 |
| 128 | PC(36:2) | 3.178039 | 1.679037 |
| 129 | ChE(20:3) | 3.189551 | 3.407779 |
| 130 | PC(36:4) | 3.18958 | 1.399946 |
| 131 | PC(40:5) | 3.195595 | 1.820181 |
| 132 | PC(34:0) | 3.196574 | 1.886997 |
| 133 | PC(30:3) | 3.203098 | 1.714828 |
| 134 | PC(30:0) | 3.203569 | 1.517133 |
| 135 | PC(38:3) | 3.22797 | 1.892619 |
| 136 | PC(42:7) | 3.233807 | 1.760342 |
| 137 | PC(34:4) | 3.238232 | 1.74204 |
| 138 | PC(38:0) | 3.266669 | 1.430291 |
| 139 | ChE(18:2) | 3.298858 | 2.545153 |
| 140 | PC(44:9) | 3.32407 | 1.557919 |
| 141 | PC(36:1) | 3.325386 | 1.813224 |
| 142 | PC(28:1) | 3.346119 | 1.473109 |
| 143 | PC(40:2) | 3.394738 | 1.740127 |
| 144 | PC(38:4) | 3.422266 | 1.398762 |
| 145 | PC(42:8) | 3.428324 | 1.566775 |
| 146 | PC(36:0) | 3.453532 | 1.587172 |
| 147 | ChE(22:5) | 3.516461 | 1.447487 |
| 148 | PC(42:6) | 3.526947 | 1.540879 |
| 149 | PC(40:0) | 3.591455 | 1.454982 |
| 150 | PC(38:1) | 3.675641 | 1.697442 |
| 151 | PC(42:2) | 3.683579 | 1.657174 |
| 152 | ChE(18:1) | 3.71797 | 2.221191 |
| 153 | PC(44:8) | 3.75485 | 1.434861 |
| 154 | ChE(16:0) | 3.930466 | 2.041653 |
| 155 | PC(40:1) | 3.960815 | 1.73677 |
| 156 | PC(44:5) | 3.979703 | 1.70252 |
| 157 | ChE(20:4) | 4.158326 | 2.722121 |
| 158 | PC(44:4) | 4.267178 | 1.416894 |
| 159 | ChE(20:5) | 4.278361 | 1.547177 |
| 160 | ChE(16:1) | 4.746785 | 1.358412 |
| 161 | dCer(d18:0-20:3) | 5.599227 | 1.727754 |

**35 Differentially regulated lipids**

| **sample** | **aged *Postn-null* / aged WT** | | |
| --- | --- | --- | --- |
| **No.** | **species** | **log2 FC** | **log10 p-value** |
| 1 | ChE(22:5) | -3.05054 | 1.377241 |
| 2 | TG(54:0)_4 | -2.92892 | 1.770527 |
| 3 | TG(54:0)_3 | -2.65853 | 1.695739 |
| 4 | ChE(20:5) | -2.64027 | 1.313075 |
| 5 | ChE(16:0) | -2.57264 | 1.755835 |
| 6 | ChE(20:4) | -2.51789 | 2.300642 |
| 7 | TG(52:7)_4 | -2.42074 | 1.884948 |
| 8 | ChE(22:6) | -2.37001 | 1.975796 |
| 9 | ChE(14:0) | -2.21091 | 1.326092 |
| 10 | ChE(18:1) | -2.17373 | 1.776412 |
| 11 | ChE(18:2) | -2.11943 | 2.092306 |
| 12 | TG(54:0)_1 | -2.0616 | 1.639871 |
| 13 | TG(52:0)_5 | -1.85288 | 1.610537 |
| 14 | ChE(22:4) | -1.82265 | 1.49584 |
| 15 | TG(48:0)_5 | -1.46656 | 1.539974 |
| 16 | TG(50:0)_4 | -1.45948 | 1.721016 |
| 17 | MG(20:2) | -1.3504 | 2.538061 |
| 18 | ChE(20:3) | -1.2748 | 1.976832 |
| 19 | MG(20:0) | -1.26493 | 1.427267 |
| 20 | PE(42:8) | -1.14418 | 1.307829 |
| 21 | Cer1P(d18:1-24:1) | -1.07405 | 2.515445 |
| 22 | ChE(18:0) | -0.97162 | 1.538075 |
| 23 | MG(22:1) | -0.92207 | 1.415102 |
| 24 | Cer1P(d18:1-20:5) | -0.84776 | 1.304533 |
| 25 | MG(20:1) | -0.84115 | 3.047315 |
| 26 | dCer(d16:0-18:4) | -0.77669 | 1.582948 |
| 27 | ChE(20:1) | -0.72606 | 2.307149 |
| 28 | Cer1P(d18:1-16:0) | -0.72164 | 1.784218 |
| 29 | LPE(22:4) | -0.71235 | 1.310247 |
| 30 | Cer1P(d18:1-18:0) | -0.69991 | 2.688684 |
| 31 | LPC(22:1) | -0.59305 | 1.605915 |
| 32 | MG(18:1) | 0.742353 | 1.996573 |
| 33 | LPE(20:5) | 0.809869 | 2.480107 |
| 34 | PE(28:5) | 0.899971 | 1.332097 |
| 35 | Cer(d14:1-22:5) | 1.456036 | 2.513761 |
